# Genetic Diversity, Population Structure and Parental Panel Optimization in Elite Popcorn (*Zea mays* L.) Inbred Lines Revealed by SSR Markers

**DOI:** 10.3390/plants15152324

**Published:** 2026-07-28

**Authors:** Dae Yeong Lee, Hye Yeong Kwon, A Jin Kim, Hee Ju Kim, Jae-Keun Choi, Ju Kyong Lee, Kyu Jin Sa

**Affiliations:** 1 Department of Crop Science, College of Ecology & Environmental Sciences, Kyungpook National University, Sangju 37224, Republic of Korea; daeyeong0218@knu.ac.kr (D.Y.L.); youngzero04@knu.ac.kr (H.Y.K.); ajin04@knu.ac.kr (A.J.K.); heeju24@knu.ac.kr (H.J.K.); 2 Maize Research Institute, Gangwon State Agricultural Research and Extension Services, Hongcheon 25160, Republic of Korea; jaekeun@korea.kr; 3 Department of Applied Plant Sciences, College of Agriculture and Life Sciences, Kangwon National University, Chuncheon 24341, Republic of Korea; jukyonglee@kangwon.ac.kr

**Keywords:** popcorn (*Zea mays* L.), SSR markers, genetic diversity, population structure, subset optimization, molecular breeding

## Abstract

This study provides a genome-wide assessment of genetic diversity, fixation status, and population structure in 77 elite Korean popcorn (*Zea mays* L.) inbred lines using 100 SSR markers. The mean number of alleles per locus was 2.99, with an expected heterozygosity (He) of 0.373 and a mean polymorphic information content (PIC) of 0.321, indicating moderate but structured diversity within a relatively narrow breeding base. The low observed heterozygosity (Ho = 0.053) and high fixation index (F = 0.875) confirmed the advanced homozygosity of these inbred lines, although residual heterozygosity ranged from 2.00% to 12.12% among lines. Population structure analyses using Bayesian STRUCTURE (*K* = 2), principal coordinate analysis (PCoA), and UPGMA clustering consistently revealed two major genetic groups with substantial admixture and hierarchical substructure. Chromosome 6 exhibited complete fixation (F = 1.000), coinciding with previously reported popping-related QTL hotspots, thereby suggesting the possible role of directional selection during elite line development. For hybrid seed purity verification, 16–35 informative codominant SSR markers were identified for each F_1_ hybrid combination, with umc2309 consistently polymorphic between the parental lines of all five tested commercial hybrid combinations. In addition, breeding-oriented subset optimization using PowerCore, CoreHunter, and their intersection retained 100% allele coverage while increasing the mean genetic distance by up to 16.0% and mean diversity indices by approximately 11%. These results demonstrate that redundancy within elite popcorn germplasm can be reduced without losing allelic representation, thereby improving parental discrimination, hybrid purity control, and breeding efficiency. Collectively, this study establishes a practical molecular framework for germplasm management, parental line discrimination, hybrid purity testing, and future popcorn breeding programs in South Korea.

## 1. Introduction

Popcorn (*Zea mays* L.) is a specialized maize type characterized by a hard endosperm and superior popping expansion. As a genetically distinct subgroup within maize germplasm, popcorn has been shaped by repeated selfing, selection for popping-related traits, and hybrid breeding programs. In South Korea, maize is cultivated primarily for food consumption and specialty markets such as waxy maize and popcorn. However, domestic popcorn production remains limited, and most popcorn products rely on imported grain, mainly from the United States. Approximately 8400 tons of popcorn grain are imported annually [1], and most popcorn products sold in theaters and retail markets are produced from imported raw materials [2]. To reduce this dependence on imports, domestic breeding programs were initiated in 1997, resulting in the development of locally adapted cultivars such as Oryun Popcorn, G-Popcorn, Oryun Popcorn 2, Kichan Popcorn, and Alchan Popcorn through conventional breeding approaches [3,4]. Although these cultivars represent significant achievements in South Korean popcorn improvement, comprehensive molecular characterization of elite parental lines remains limited. This lack of genetic information constrains evidence-based parental selection, genetic purity assessment, and efficient utilization of breeding resources. Therefore, genome-wide molecular profiling using simple sequence repeat (SSR) markers provides a valuable approach for quantifying allelic diversity, identifying genetic redundancy, and supporting parental-line selection, hybrid purity testing, and germplasm management in breeding programs [5].

SSR markers have been widely applied to assess genetic diversity and population structure in maize [6,7]. Because of their codominant inheritance and high polymorphism, SSRs enable reliable estimation of allele frequencies, heterozygosity, and population differentiation [8,9]. Numerous SSR-based studies have demonstrated substantial allelic variation within maize inbred line collections. For example, a study of 30 maize inbred lines genotyped with 100 SSR markers showed 5.50 alleles per locus, a polymorphism information content (PIC) value of 0.624, and gene diversity of 0.671 [10]. Similarly, analysis of 79 Korean popcorn inbred lines using 50 SSR markers identified 395 alleles, averaging 7.9 alleles per locus, with relatively high genetic diversity (mean GD = 0.644) and PIC (0.602) [11]. Other studies analyzing maize landraces and breeding populations have reported diversity levels ranging from 4.72 alleles per locus and PIC values around 0.51, depending on germplasm origin and marker selection [12]. These findings highlight that diversity estimates vary substantially with breeding history, geographic origin, marker sets, and sampling strategies, emphasizing the importance of population-specific SSR profiling for accurate interpretation of diversity parameters and meaningful comparisons among breeding materials [13].

Beyond diversity estimation, genome-wide SSR data provide a basis for inferring population structure and evolutionary relationships. Bayesian model-based clustering, particularly the STRUCTURE algorithm, is widely used to identify discrete genetic groups and detect cryptic admixture among individuals [14]. Many studies apply a stringent membership threshold (typically Q ≥ 0.80) to assign individuals to groups, classifying lower values as admixed. Because a single method may overlook complex relationships, distance-based approaches such as UPGMA or neighbor-joining are often used to provide a hierarchical view of genetic proximity and are commonly interpreted alongside multivariate ordination methods such as principal coordinate analysis (PCoA) [15]. The combined use of STRUCTURE, PCoA, and UPGMA thus offers a robust framework for evaluating population structure and guiding parental selection and germplasm management.

Another important parameter in inbred line evaluation is residual heterozygosity (RH), which directly affects hybrid seed purity and genetic stability. Advanced maize inbred lines are expected to exhibit very low observed heterozygosity; for example, SSR-based analyses have reported values as low as 0.017 in maize inbred line panels [10]. Genetic purity studies further indicate that parental inbred lines should ideally maintain RH below 5%, whereas higher values may reflect incomplete fixation [16,17]. Monitoring RH is therefore essential for ensuring genetic uniformity, maintaining hybrid seed purity, and supporting stable seed production in commercial breeding programs.

In addition to diversity and population structure analyses, efficient utilization of elite germplasm requires strategically optimized representative subsets that retain complete allelic representation while minimizing redundancy and enhancing genetic contrast among parental lines [5]. PowerCore implements the Advanced M (Maximization) strategy to preserve all alleles present in the original population within a reduced sample size [18]. CoreHunter further refines subset selection through multi-objective optimization that balances allelic richness and genetic distance to generate representative and genetically diverse panels [19]. These approaches enable the construction of practical breeding panels that maintain rare alleles while improving operational efficiency and parental discrimination.

Despite extensive SSR-based studies in maize, most previous investigations have focused primarily on broad germplasm collections, landrace populations, or general diversity assessments. In contrast, integrated analyses combining genome-wide diversity assessment, population structure inference, residual heterozygosity evaluation, parental-line discrimination marker selection, and breeding-oriented subset optimization remain limited in elite popcorn inbred-line collections directly associated with commercial hybrid breeding programs. The novelty of this study lies not in the application of individual analytical methods, which are well established, but in their integration within a well-defined Korean elite popcorn breeding population representing the parental foundation of commercially developed F_1_ hybrids. Therefore, the present study aimed to (i) evaluate SSR-based genetic diversity and residual heterozygosity in 77 Korean popcorn inbred lines to assess their genetic fixation status, (ii) infer population structure and genetic relationships using complementary approaches, including STRUCTURE, PCoA, and UPGMA analyses, (iii) identify informative SSR markers for parental-line discrimination and F_1_ hybrid purity verification, and (iv) establish an optimized representative subset that preserves complete allelic coverage while enhancing breeding efficiency in elite popcorn germplasm. Unlike previous studies that primarily emphasized genetic diversity estimation, the objective of the present study was to establish a practical molecular framework for parental-line management, hybrid seed purity testing, fixation assessment, and breeding-panel optimization in Korean popcorn breeding. The resulting dataset and optimized parental panels provide valuable resources for germplasm management and serve as a foundation for future genomic studies, including QTL mapping, genome-wide association studies, genomic selection, and superior allele discovery in popcorn.

## 2. Results

### 2.1. Genetic Diversity, Fixation Level, and Informative SSR Markers

A total of 100 SSR markers were used to evaluate genetic diversity in 77 popcorn inbred lines. Across loci, the mean number of alleles per locus (Na) was 2.99, with a mean effective number of alleles (Ne) of 1.805 and a Shannon’s diversity index (I) of 0.635. The overall expected heterozygosity (He) was 0.373, whereas observed heterozygosity (Ho) was markedly lower (0.053), reflecting the highly homozygous nature of these inbred lines. The mean major allele frequency (MAF) was 0.715, the mean polymorphism information content (PIC) was 0.321, and the fixation index (F) averaged 0.875, indicating strong inbreeding across the collection (Table 1).

Genetic diversity varied considerably among chromosomes. Chromosome 4 exhibited the highest diversity (Na = 3.22; Ne = 2.132; I = 0.808; He = 0.458; PIC = 0.408), whereas chromosome 6 displayed the lowest diversity (Na = 2.63; Ne = 1.440; I = 0.458; He = 0.265; PIC = 0.227) and reached complete fixation (F = 1.000). At the locus level, polymorphism ranged widely: umc1995 displayed exceptionally high genetic variability (Na = 7; Ne = 4.661; PIC = 0.753), whereas umc1197 was monomorphic (Na = 1; PIC = 0). Several loci exhibited negative F values, indicating locus-specific excess heterozygosity. This pattern may reflect sampling variation, residual heterozygosity, incomplete fixation during inbred line development, or occasional outcrossing or seed mixture (Table S1).

Residual heterozygosity (RH) further highlighted variation in fixation levels among lines. Across the 77 accessions, RH ranged from 2.00% to 12.12%. Inbred lines such as 21PS8142 (2.00%) and 21PS8113 (2.08%) were nearly completely fixed, whereas 20PS8118 (11.46%) and 21PS8043 (12.12%) retained comparatively high residual heterozygosity. Among the seven parental lines, RH ranged from 4.00% (GP3) to 7.45% (GP2), indicating generally acceptable fixation levels, with moderate residual segregation in some cases (Table 2 and Table S2).

Polymorphic SSR markers that could distinguish the parental lines were identified for five F_1_ hybrid combinations. The number of informative markers varied among hybrid pairs, with 22 markers identified for Oryun Popcorn (GP1 × GP2), 34 for G-Popcorn (GP3 × GP4), 16 for Oryun Popcorn 2 (GP5 × GP3), 35 for Kichan Popcorn (GP6 × GP3), and 30 for Alchan Popcorn (GP2 × GP7). Each hybrid combination possessed a distinct set of codominant markers suitable for hybrid purity verification. Notably, umc2309 was polymorphic across all five hybrid combinations, indicating its potential as a universal marker for parental-line discrimination. Furthermore, bnlg1138, umc1051, and bnlg1036 were informative across multiple hybrid combinations, supporting their utility for routine F_1_ seed purity testing (Table 3).

### 2.2. Genetic Structure Revealed by STRUCTURE, PCoA, and UPGMA Analyses

**Figure 1:**
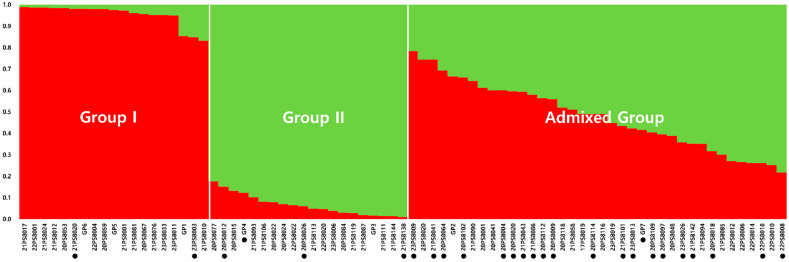
Population structure of 77 popcorn inbred lines inferred by STRUCTURE analysis based on 100 SSR markers at *K* = 2. Each vertical bar represents an individual accession, and the proportion of each color indicates the estimated membership coefficient (Q) for the corresponding genetic group. Black circles denote the accessions included in the optimized intersection subset selected by both PowerCore and CoreHunter.

Population structure analysis using STRUCTURE (*K* = 2) identified two primary genetic groups (Group I and Group II), along with a subset of admixed accessions (Figure S1). Based on a membership coefficient threshold of Q ≥ 0.80, individuals were classified into discrete genetic backgrounds or as admixed. Of the 77 inbred lines, 19 (24.7%) were assigned to Group I and 20 (26.0%) to Group II, and the remaining 38 accessions (49.3%) were classified as admixed (Figure 1; Table 4).

**Figure 2:**
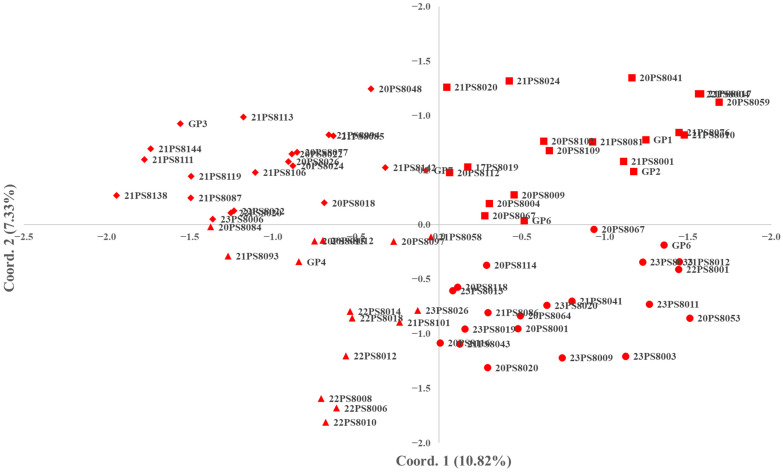
Principal coordinate analysis (PCoA) of 77 popcorn inbred lines based on a genetic distance matrix using 100 SSRs. The first and second principal coordinates explained 10.82% and 7.33% of the total molecular variation, respectively. Different symbols indicate PCoA quadrant groups: squares, diamonds, triangles, and circles represent quadrants I, II, III, and IV, respectively.

Principal coordinate analysis (PCoA), based on the genetic distance matrix generated in GenAlEx, further revealed the distribution of the 77 inbred lines. The first and second principal coordinates explained 10.82% and 7.33% of the total variation, respectively (18.14% cumulative). According to their positions in the quadrants defined by these axes, 20 accessions were located in Quadrant I, 21 in Quadrant II, 15 in Quadrant III, and 21 in Quadrant IV (Figure 2; Table 4). STRUCTURE Group I accessions were mainly distributed in Quadrants I and IV, whereas Group II accessions were predominantly located in Quadrants II and III. Admixed accessions were dispersed across all quadrants.

**Figure 3:**
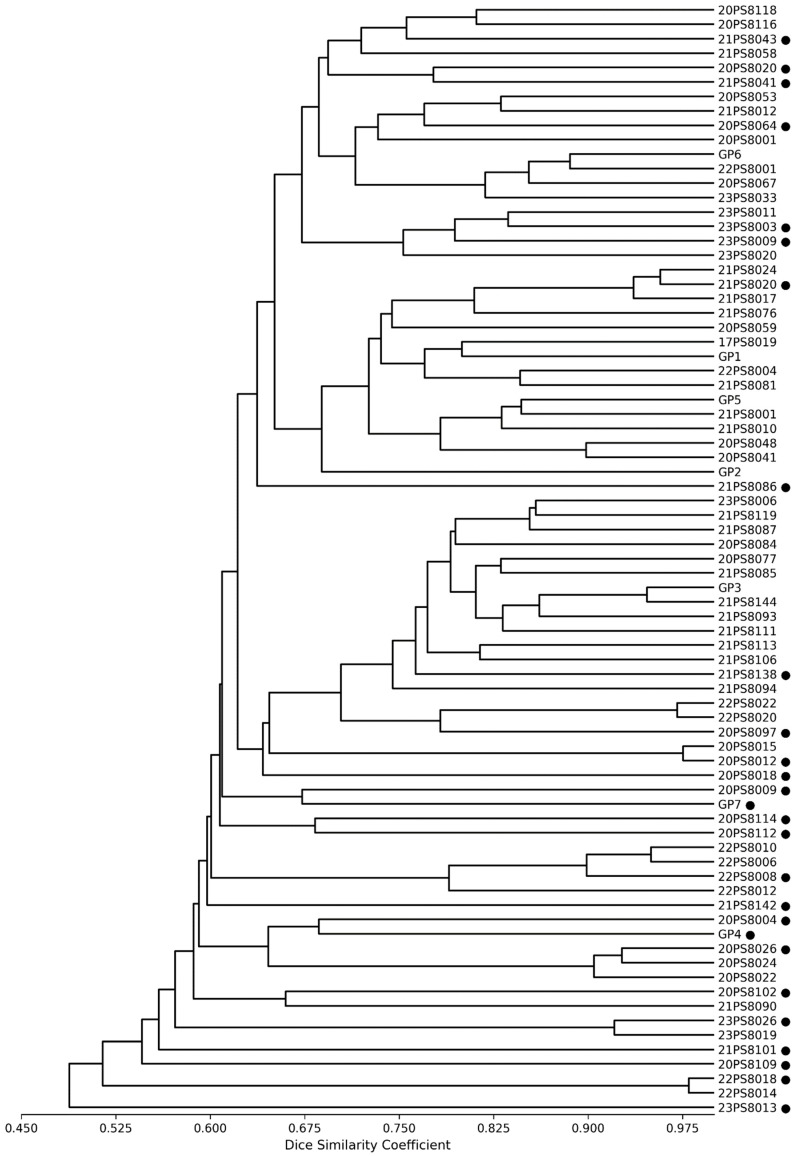
UPGMA dendrogram of 77 popcorn inbred lines constructed using Dice similarity coefficients based on 100 SSR markers. Major clusters were defined at a similarity threshold of 0.621, and the largest cluster was further subdivided at 0.70 (see Table 4). Black circles indicate accessions included in the optimized intersection subset selected by both PowerCore and CoreHunter.

Hierarchical clustering using UPGMA, based on Dice similarity coefficients, partitioned the 77 inbred lines into 12 major clusters at a similarity threshold of 0.621. The largest cluster (Cluster 1) comprised 54 accessions (70.1%), while the remaining 23 accessions were distributed among clusters 2 to 12. Further subdivision of Cluster 1 at a similarity level of 0.70 resolved ten distinct subgroups. Subgroup 1-I contained 17 accessions, followed by 1-II (14 accessions) and 1-III (8 accessions). Subgroups 1-IV and 1-V each contained four accessions, whereas 1-VI and 1-VII comprised two accessions each. The smallest subgroups (1-VIII, 1-IX, and 1-X) were represented by a single accession. STRUCTURE Group II accessions were predominantly assigned to subgroup 1-I, whereas Group I accessions were mainly distributed in subgroups 1-II and 1-III. Minor clusters (2–12) contained between one and five accessions (Figure 3; Table 4).

### 2.3. Breeding Panel Optimization and Methodological Concordance

**Figure 4:**
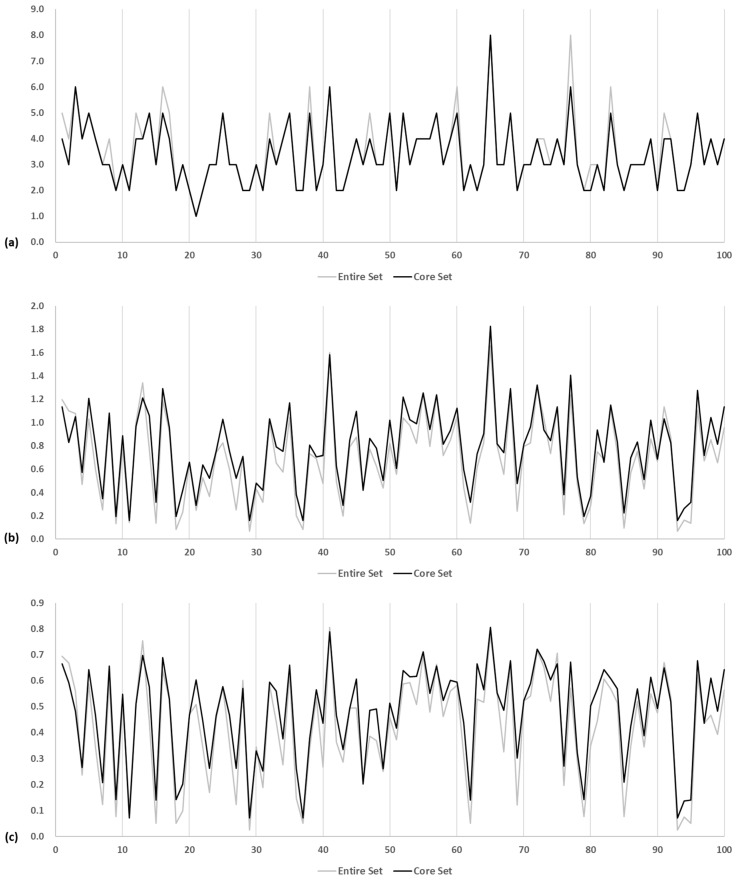
Comparison of genetic diversity parameters between the full collection (77 inbred lines) and the optimized breeding panel across 100 SSR loci. Panels (**a**), (**b**), and (**c**) represent allele number, Shannon’s information index, and Nei’s gene diversity, respectively. Grey lines correspond to the full collection, whereas black lines denote the optimized panel.

To assess breeding-oriented subset optimization strategies, three representative panels, PowerCore (36 accessions), CoreHunter (36 accessions), and their intersection subset (27 accessions), were compared with the full collection (77 accessions). All optimized panels retained 100% allelic coverage, indicating complete preservation of the alleles detected in the entire dataset (Figure 4; Table 5).

Mean pairwise genetic distance increased from 0.393 in the full collection to 0.430 in PowerCore, 0.450 in CoreHunter, and 0.456 in the intersection subset. Relative to the entire collection, mean pairwise genetic distance increased by 9.5%, 14.5%, and 16.0%, respectively (Table 5). Similarly, mean expected heterozygosity (He) increased from 0.373 in the full set to 0.396 (PowerCore), 0.414 (CoreHunter), and 0.415 (intersection subset), while mean PIC values increased from 0.321 to 0.341, 0.357, and 0.357, respectively (Table 5). These results demonstrate that redundancy within the full elite collection can be reduced while simultaneously enhancing genetic contrast among selected parental lines.

**Figure 5:**
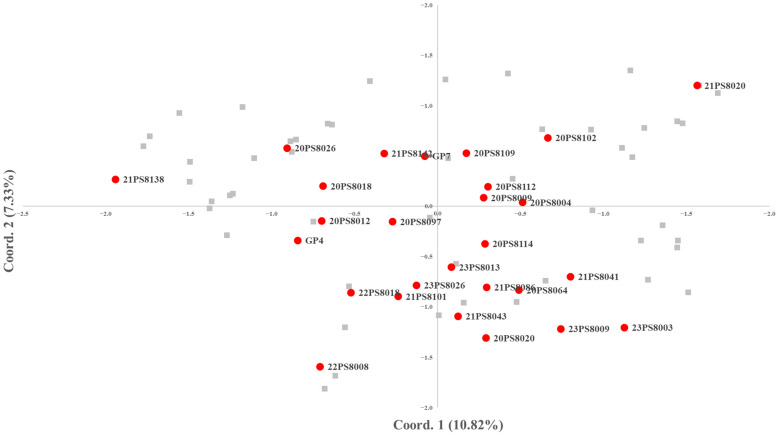
Principal coordinate analysis (PCoA) showing the distribution of the optimized representative subset within the genetic structure of 77 popcorn inbred lines based on 100 SSR markers. Red circles denote accessions included in the optimized intersection subset selected by both PowerCore and CoreHunter, whereas grey squares represent the remaining lines.

At the locus level, diversity values across the 100 SSR markers in the optimized panels were largely comparable with those of the full set, with several loci showing equal or higher He and PIC values in CoreHunter and the intersection subset (Figure 4). Notably, the intersection subset, comprising only 27 accessions (35.1% of the entire collection), retained complete allelic representation while exhibiting the highest mean genetic distance. Across diversity indices and clustering patterns, the optimized panels preserved the overall genetic structure of the 77 inbred lines (Figure 4 and Figure 5; Table 5), confirming their suitability as streamlined breeding panels for efficient parental management.

## 3. Discussion

This study presents a comprehensive molecular characterization of elite Korean popcorn inbred lines that serve as parental materials for commercial hybrid development. Although SSR-based diversity and population structure analyses are well-established approaches, the integration of genome-wide diversity assessment, residual heterozygosity evaluation, parental-line discrimination, hybrid purity marker development, and breeding-oriented subset optimization within a single elite breeding population has rarely been reported in popcorn germplasm. By combining these complementary analyses, the present study extends beyond conventional diversity surveys and provides practical molecular tools directly applicable to parental-line management, hybrid seed quality control, and germplasm optimization in Korean popcorn breeding programs. Importantly, the present study bridges the gap between conventional molecular characterization and practical breeding implementation by providing molecular resources directly applicable to commercial breeding programs. The resulting dataset and optimized breeding panels provide valuable resources for the efficient utilization of elite germplasm and establish a foundation for future genomic studies, including QTL mapping, genome-wide association studies, genomic selection, and superior allele discovery. Furthermore, the molecular resources developed in this study can be immediately implemented in commercial popcorn breeding programs for parental-line identification, hybrid seed quality control, and efficient management of breeding populations. Therefore, the practical value of the present work extends beyond conventional diversity assessment and provides direct support for modern popcorn breeding and germplasm management. Unlike previous popcorn diversity studies that primarily focused on germplasm characterization, the present study directly links molecular characterization to breeding implementation by providing validated tools for parental-line management, fixation assessment, hybrid seed purity testing, and breeding-panel optimization.

### 3.1. Genome-Wide Genetic Diversity and Breeding Bottlenecks in Korean Popcorn Inbred Lines

In the present Korean popcorn inbred line collection, the mean number of alleles per locus (Na = 2.99), expected heterozygosity (He = 0.373), and mean PIC value (0.321) indicate a moderately polymorphic yet genetically structured parental pool (Table 1 and Table S1). Although measurable variation remains, these diversity estimates are substantially lower than those reported for maize panels derived from broader and more heterogeneous germplasm sources. For example, a tropical maize inbred line panel genotyped with 100 SSR markers exhibited a mean of 5.50 alleles per locus, a PIC value of 0.624, and gene diversity of 0.671 [10], reflecting the inclusion of genetically diverse introductions and a less restrictive selection history. Likewise, a Korean genebank collection comprising 105 maize accessions revealed exceptionally high allelic richness (1104 alleles across 100 SSR loci; mean Na = 11.0) and elevated diversity indices (mean GD = 0.73; mean PIC = 0.70) [20], consistent with the broad genetic representation typically preserved in ex situ germplasm resources. The contrast becomes even more pronounced in landrace-based studies. A South Sudan landrace panel (n = 37) characterized using 27 SSR markers detected 200 alleles (mean 7.4 alleles per locus) and high marker informativeness (mean PIC = 0.699), accompanied by substantial heterozygosity (Ho = 0.351) [21]. Similarly, a race-level survey of maize populations from Chiapas, Mexico (73 populations; 31 SSR loci) reported 787 alleles with an average of 25.39 alleles per locus and high expected heterozygosity (He = 0.677), illustrating the extensive allelic reservoir retained in traditional maize races and related materials [13]. Together, these comparisons indicate that genebank and landrace populations retain broader allelic diversity due to heterogeneous ancestry, farmer-mediated seed exchange, limited directional selection, and weaker historical bottlenecks.

Although a previous study of Korean popcorn inbred lines reported comparatively high polymorphism (mean 7.9 alleles per locus; GD = 0.644; PIC = 0.602) [13], the lower diversity observed in the present study likely reflects differences in breeding stage rather than inherent limitations of popcorn germplasm. The materials analyzed in this study mainly consist of elite inbred lines used as hybrid parents, which have undergone repeated selfing and strong directional selection during the breeding process, resulting in reduced genetic variation. In addition, diversity estimates can be influenced by the type, number, and genomic distribution of the SSR markers employed, which may partially explain the discrepancies between the two studies. Nevertheless, the earlier panels may have included materials at intermediate stages of selection, encompassing broader genetic backgrounds prior to intensive purification. In contrast, the current collection consists of elite inbred lines used as parents of registered commercial hybrids. These lines have undergone repeated cycles of selfing and strong directional selection for key agronomic and quality traits, including popping expansion, kernel hardness, plant architecture, and local adaptation. Repeated selfing progressively reduces heterozygosity across generations [22], while directional selection promotes allele fixation and selective sweeps that diminish genetic diversity [23]. Accordingly, the relatively low Na (2.99) and PIC (0.321) observed in this study are consistent with intensive selection within a narrow genetic base and repeated founder use during elite line development [24,25]. Collectively, these findings indicate that the analyzed Korean popcorn breeding materials constitute a relatively narrow genetic pool with a high degree of fixation. Consequently, they exhibit substantial genetic uniformity but lower genetic diversity than earlier-stage breeding materials, landrace populations, or genebank collections. From a breeding perspective, this pattern suggests that, while directional selection has improved popping-related performance, the limited allelic reservoir may constrain future gains for additional targets such as stress tolerance, disease resistance, and yield stability [24]. To address this limitation, incorporating genetically divergent donor materials (e.g., selected genebank accessions or landrace-derived sources) through pre-breeding, followed by backcrossing or recurrent selection, could broaden the elite germplasm pool while maintaining desirable popcorn-type kernel traits [13,20,24]. Furthermore, expanding the breeding panel with additional domestic and exotic popcorn germplasm would help broaden the Korean breeding base and facilitate the establishment of more distinct heterotic pools for long-term hybrid improvement [23,24].

### 3.2. Chromosome-Specific Fixation and Putative Selection Signals on Chromosome 6

Genetic diversity was not uniformly distributed across chromosomes. Chromosome 4 exhibited the highest diversity parameters (He = 0.458; PIC = 0.408), whereas chromosome 6 showed markedly reduced diversity and complete fixation (F = 1.000) (Table 1 and Table S1). This chromosomal heterogeneity suggests that different genomic regions may have experienced distinct evolutionary histories, breeding bottlenecks, or selection pressures during elite line development. Similar chromosome-specific patterns of diversity have been reported in maize inbred panels, where particular genomic regions retained either elevated polymorphism or reduced variation depending on breeding history and selection intensity [10]. The complete fixation observed on chromosome 6 is particularly noteworthy. Although QTL mapping was not conducted in the present study, a comprehensive meta-QTL analysis integrating multiple popcorn studies identified a major QTL hotspot on chromosome 6, particularly within bin 6.06–6.07, where several QTLs associated with popping-related traits have been reported [26]. Therefore, the reduced diversity observed on chromosome 6 may reflect historical selection for favorable alleles contributing to popping performance, kernel quality, or other agronomically important characteristics during the development of Korean elite popcorn inbred lines. Nevertheless, this interpretation should be considered with caution. Because only eight SSR markers were available on chromosome 6, the observed complete fixation may partially reflect limited marker coverage rather than a true chromosome-wide selective sweep. Consequently, the observed pattern should be regarded as a putative rather than definitive signature of selection, and the current results do not allow clear discrimination between strong directional selection and reduced detectable variation resulting from marker-density limitations. Despite this limitation, the chromosome-wide reduction in diversity remains consistent with the expectation that repeated selfing and long-term selection can promote allele fixation within elite breeding populations. From a breeding perspective, the extensive fixation observed on chromosome 6 may indicate that favorable alleles have already been largely stabilized within the current parental pool, thereby contributing to the genetic uniformity and phenotypic consistency required for commercial hybrid production. However, it may also suggest a limited reservoir of genetic variation available for future genetic improvement in this genomic region. To determine whether the observed pattern reflects a genuine selection signature, future studies employing higher-density genotyping approaches (e.g., SNP arrays or sequencing-based methods) will be necessary to refine the boundaries of the putative sweep and to detect fine-scale genetic variation that may not have been captured by the current SSR marker set [7,9,23,25,26].

### 3.3. Residual Heterozygosity and Implications for Hybrid Seed Purity

The fixation status of the collection further confirms its advanced inbred line nature. The low observed heterozygosity (Ho = 0.053) and high fixation index (F = 0.875) indicate strong homozygosity across the 77 inbred lines (Table 1 and Table S1), consistent with expectations for stabilized maize inbred lines, in which heterozygosity progressively declines through successive selfing. Notably, despite the overall high level of fixation, certain loci exhibited negative F values (e.g., −0.553 on chromosome 1). This localized excess of heterozygotes suggests that specific genomic regions in these popcorn lines may resist complete fixation during inbreeding, potentially due to biological constraints or selection pressures. Such residual heterozygosity (RH) at specific loci contributes to the overall variation in RH observed within the collection. Nevertheless, RH value within the collection ranged from 2.00% to 12.12%, whereas parental lines used in commercial hybrids exhibited RH values between 4.00% and 7.45% (Table 2 and Table S2). In maize breeding programs, RH levels below 5% are generally recommended for parental inbred lines to ensure hybrid seed purity and uniform agronomic performance. However, because maize is a naturally cross-pollinated species, residual heterozygosity of up to 10% may occasionally persist even in advanced inbred lines. Despite this biological tendency, maintaining RH below 5% is widely considered desirable for commercial seed production in order to minimize genetic impurity and ensure phenotypic stability [16,17,27]. Therefore, although most Korean popcorn parental lines fall within or close to the recommended fixation thresholds, several lines exceeding 7% RH suggest that additional purification cycles may further enhance genetic stability. Routine SSR-based monitoring of fixation status will remain important for maintaining hybrid quality and improving breeding efficiency. Variation in RH among lines may reflect differences in the number of selfing generations, residual segregating segments, or occasional contamination during seed increase. For lines exceeding the preferred RH threshold, one to two additional generations of strict selfing under isolation with single-plant selection could improve uniformity, while routine marker-based identity checks at breeder or foundation seed stages may help prevent genetic drift and seed mixture during long-term multiplication [16,17].

### 3.4. Highly Informative SSR Markers for Hybrid Purity Verification

A major applied contribution of this study is the identification of codominant SSR markers capable of discriminating each parental combination used in commercial F_1_ hybrids. Across the five hybrid combinations examined, 16–35 informative markers were identified per cross, providing robust molecular tools for hybrid identification and seed purity assessment (Table 3). Among these markers, umc2309 was consistently polymorphic across all five hybrid combinations, indicating that it is a highly informative marker for hybrid identification and genetic purity verification. Such cross-combination markers are particularly valuable for breeding programs that require routine genetic purity testing and accurate parent-offspring verification. Molecular quality-control systems based on SSR or SNP markers are now widely adopted in maize breeding programs [17], and the present results establish a dedicated marker set specifically applicable to Korean popcorn hybrids. The identified marker sets can support reliable seed certification, strengthen intellectual property protection, and enhance the overall integrity and efficiency of commercial popcorn breeding programs. Although 16–35 informative markers were identified for each hybrid combination, routine quality assurance (QA) or quality control (QC) procedures can be streamlined by employing a smaller tiered panel that prioritizes loci with clear codominant banding patterns, distinct parental alleles, and consistent amplification performance. In this framework, umc2309 can function as an anchor marker across hybrids, supplemented by a minimal set of cross-specific markers. Compared with conventional grow-out tests, marker-assisted purity testing can substantially reduce the time, labor, and costs required for hybrid seed quality assessment. Therefore, the marker sets identified in this study provide practical tools for rapid, reliable, and cost-effective quality-control procedures in commercial popcorn seed production. In the longer term, these SSR-based assays may be further complemented or replaced by SNP-based platforms to enable higher-throughput genotyping [7,17].

### 3.5. Integrated Population Structure and Fine-Scale Hierarchical Clustering

Bayesian population structure analysis (*K* = 2) identified two major genetic groups, with approximately half of the accessions classified as admixed (Figure 1; Table 4). The relatively high proportion of admixed lines (49.3%) indicates historical recombination among partially overlapping breeding backgrounds rather than the presence of strictly isolated heterotic pools. Previous SSR studies of popcorn have also reported multiple genetic groups with substantial admixture [11], suggesting that popcorn breeding programs frequently rely on recurrent recombination among related inbred line sources. Principal coordinate analysis (PCoA) further supported this pattern, with the first two axes explaining 18.14% of the total molecular variation. Although this proportion appears modest, comparable levels of explained variation are commonly reported in maize SSR-based PCoA analyses [10]. Notably, the quadrant distribution in the PCoA plot broadly corresponded with STRUCTURE group assignments, demonstrating consistency between model-based and distance-based approaches (Table 4). Hierarchical clustering using the UPGMA method resolved the 77 inbred lines into 12 major clusters at a Dice similarity threshold of 0.621. The largest cluster (Cluster 1) encompassed 70.1% of the accessions; however, further subdivision at a similarity threshold of 0.70 revealed ten subgroups, indicating substantial fine-scale genetic structure within the primary cluster. Similar nested hierarchical patterns have been observed in several maize inbred line collections, suggesting that such fine-scale structuring is a common feature of advanced breeding materials [28,29]. Overall, the convergence of STRUCTURE, PCoA, and UPGMA results reinforces the robustness of the inferred genetic architecture. The integrated application of these complementary analytical approaches provides a reliable framework for heterotic grouping and informed parental selection in popcorn breeding programs. From a practical standpoint, the two-group pattern can serve as an initial framework for testcross design: crosses between divergent parents (e.g., between STRUCTURE Groups I and II and/or among distant UPGMA subgroups) can be prioritized, whereas highly similar parental pairs may be deprioritized to reduce redundancy. However, because many lines exhibit admixed ancestry, molecular grouping alone may not fully predict heterosis. Therefore, combining ability tests, together with key agronomic traits such as popping expansion, flake type, yield, and lodging tolerance, should be integrated to establish stable heterotic pools [7,30]. Furthermore, integrating molecular grouping with comprehensive phenotyping (e.g., popping expansion, flake morphology, kernel hardness, yield stability, and stress tolerance) will be essential for translating genetic structure into predictable breeding performance.

### 3.6. Breeding-Oriented Subset Optimization and Redundancy Reduction

Efficient utilization of germplasm resources is a central objective in modern breeding programs. In this study, all three subset optimization strategies (PowerCore, CoreHunter, and the intersection subset) retained 100% of the alleles present in the entire collection. Notably, the intersection subset, comprising only 27 accessions (35.1% of the original population), preserved complete allelic representation while maximizing genetic distance and diversity indices. Compared with the full collection, the intersection subset showed a 16.0% increase in mean pairwise genetic distance, while mean expected heterozygosity (He) and PIC values increased by approximately 11%. These results demonstrate that substantial redundancy within the original collection can be effectively reduced without compromising genetic diversity. These results are consistent with the rationale of core collection approaches, which aim to reduce redundancy and improve manageability while preserving as much of the collection’s genetic diversity as possible [5]. The ability to reduce the collection size by nearly two-thirds while preserving full allelic representation has significant implications for breeding panel design, parental selection efficiency, and resource-efficient breeding management. Because the intersection subset retains full allelic coverage while maximizing genetic distance, it can be prioritized as an operational panel for multi-environment phenotyping and parental management. At the same time, non-core accessions should be maintained as reserve germplasm, as SSR-similar lines may still differ at untyped loci or carry favorable combinations. Therefore, core subsets should be regarded as management tools rather than replacements for the full germplasm collection [5].

### 3.7. Implications for Elite Popcorn Breeding Programs

Although numerous SSR-based studies have examined genetic diversity and population structure in maize, relatively few have integrated genome-wide diversity assessment, fixation analysis, hybrid purity marker development, population structure characterization, and breeding-oriented subset optimization within a single elite popcorn breeding population. The present study addresses this gap by combining comprehensive genetic diversity characterization with direct applications for breeding. The structured genetic diversity observed in Korean popcorn inbred lines, together with acceptable fixation levels and effective redundancy reduction through optimized panel selection, provides a robust foundation for expanding heterotic divergence, improving hybrid performance, and enhancing breeding efficiency in elite popcorn programs. Collectively, these findings provide both theoretical insights into the genetic architecture of Korean popcorn germplasm and practical molecular tools to enhance breeding precision, parental discrimination, and hybrid seed quality assurance. Importantly, the molecular resources developed in this study can be immediately implemented in commercial popcorn breeding programs for parental-line identification, hybrid seed quality control, and efficient management of breeding populations. Compared with conventional grow-out tests, marker-assisted purity testing can substantially reduce the time, labor, and costs required for hybrid seed quality assessment. From a practical breeding perspective, the selected SSR markers can be implemented as routine QA/QC tools in breeder and foundation seed production, while testcrosses between genetically divergent parental lines can be prioritized for hybrid development and subsequently validated through combining ability and multi-environment trials. In addition, the 27-line intersection subset may serve as an operational diversity panel for phenotyping, parental selection, and germplasm management, and future genomic studies.

### 3.8. Study Limitation and Furture Perspectives

Several limitations should be acknowledged. First, the present study was based on SSR markers, which provide lower genome coverage than contemporary SNP-based genotyping platforms. Although SSR markers remain highly informative for diversity assessment, parental-line discrimination, and hybrid purity testing, they may not fully capture fine-scale genomic variation. Second, the analyzed materials represent elite Korean popcorn breeding germplasm and therefore may not encompass the full extent of global popcorn diversity. Nevertheless, these lines constitute the parental foundation of currently utilized Korean commercial popcorn hybrids and are therefore highly relevant for domestic breeding applications. Future studies integrating broader international germplasm collections and high-density genomic datasets will enable finer-resolution analyses of population structure, selection signatures, heterotic patterns, and genomic regions associated with economically important popcorn traits. Furthermore, the molecular framework established in this study provides a valuable foundation for future QTL mapping, genome-wide association studies (GWAS), genomic selection, and superior allele discovery in popcorn. Such efforts will further strengthen genomics-assisted breeding and facilitate the development of improved popcorn cultivars.

## 4. Materials and Methods

### 4.1. Plant Materials and DNA Extraction

A total of 77 popcorn inbred lines developed and maintained at the Maize Experiment Station, Gangwon Agricultural Research and Extension Service, Hongcheon, Republic of Korea, were used in this study (Table 4). These inbred lines are used as parental materials for the production of five F_1_ hybrid cultivars (Table 3). Specifically, lines designated as GP1–GP7 serve as parental lines for the commercial hybrids Oryun Popcorn, G-Popcorn, Oryun Popcorn 2, Kichan Popcorn, and Alchan Popcorn in South Korea. These hybrids have been officially registered with the Korea Seed & Variety Service (KSVS) and are currently cultivated in South Korea. Detailed information on the pedigree sources, breeding generations, and breeding backgrounds of the 77 inbred lines, including the GP1–GP7 parental groups, is provided in Supplementary Table S3. Genomic DNA was extracted from young leaf tissue using a modified cetyltrimethylammonium bromide (CTAB) method [31].

### 4.2. SSR Genotyping

A total of 100 simple sequence repeat (SSR) markers, evenly distributed across the ten maize chromosomes, were selected to ensure genome-wide coverage (Table S1). Information on marker chromosomal positions and primer sequences was obtained from MaizeGDB (https://www.maizegdb.org/; accessed on 26 March 2025).

PCR amplification was performed in a 20 μL reaction volume containing genomic DNA, primers, dNTPs, buffer, and Ex Taq polymerase (Takara Bio Inc., Shiga, Japan). The thermal cycling conditions consisted of an initial denaturation at 94 °C, followed by touchdown PCR with annealing temperatures gradually decreasing from 65 °C to 55 °C, and a final extension step at 72 °C. PCR products were resolved on 6% polyacrylamide gels in 0.5× Tris–borate–EDTA (TBE) buffer using a vertical electrophoresis system (C.B.S. Scientific, Del Mar, CA, USA) and visualized by ethidium bromide staining.

### 4.3. Data Analyses

Fragments amplified using the SSR primers were scored as codominant genotypes based on fragment length polymorphism. Genetic diversity parameters, including the number of alleles (Na), effective number of alleles (Ne), Shannon’s information index (I), observed heterozygosity (Ho), expected heterozygosity (He), polymorphism information content (PIC), major allele frequency (MAF) and fixation index (F), were calculated using GenAlEx v6.5 [32] and PowerMarker v3.25 [33]. Pairwise genetic similarity among parental lines was estimated using the Dice similarity coefficient implemented in PAST v5.3. An unweighted pair group method with arithmetic mean (UPGMA) dendrogram was constructed from the resulting similarity matrix using PAST v5.3 [34]. Major clusters were defined at a Dice similarity threshold of 0.621. To further resolve hierarchical structure within the largest cluster, an additional subdivision was performed at a similarity level of 0.70. Subgroups within the primary cluster were designated sequentially with Roman numerals (e.g., 1-I to 1-X; see Table 4) according to cluster size in descending order, whereas minor clusters identified at the 0.621 threshold were assigned numeric labels (e.g., 2–12; see Table 4).

Population structure was inferred using STRUCTURE v2.3.4 under an admixture model with *K* values ranging from 1 to 10 [14]. For each *K* value, five independent runs were performed, with a burn-in period of 100,000 iterations followed by 100,000 Markov chain Monte Carlo (MCMC) iterations. The optimal number of subpopulations was determined using the Δ*K* method [35]. Individual accessions were assigned to a specific group when the membership coefficient (Q) was ≥0.80; otherwise, they were classified as admixed. Principal coordinate analysis (PCoA) was performed in GenAlEx v6.5 to visualize genetic relationships among the inbred lines based on the genetic distance matrix calculated from the SSR data. The analysis was conducted using a covariance matrix with data standardization. Accessions were assigned to one of four PCoA clusters according to their positions within the quadrants defined by the first two principal coordinate axes (Coord. 1 and Coord. 2). This classification was subsequently compared with the STRUCTURE and UPGMA clustering results to evaluate concordance among methods.

For hybrid purity assessment, SSR markers were screened separately for each F_1_ parental combination. A marker was considered informative when it satisfied the following criteria: (i) both parental lines were homozygous and exhibited reproducible amplification profiles; (ii) the parental alleles displayed clearly distinguishable fragment-size polymorphisms, allowing reliable discrimination between the two parents; (iii) amplification profiles were sharp, unambiguous, and consistently scorable, with no evidence of stutter bands, weak amplification, non-specific products, or missing data in either parent; and (iv) the expected F_1_ genotype could be unequivocally identified as a codominant heterozygous profile containing both parental alleles. PowerCore v2 [18] was run using the Advanced M strategy with the objective of retaining all alleles detected in the full collection while minimizing redundant accessions. No prior population structure, PCoA, or UPGMA groupings was imposed during subset selection. CoreHunter v3.2 [19] was run using the same SSR marker dataset, and the target subset size was fixed at 36 accessions to allow direct comparison with the PowerCore output. CoreHunter optimization was performed by prioritizing allelic representation and mean pairwise genetic distance among selected accessions. The intersection subset was defined as the set of accessions commonly selected by both PowerCore and CoreHunter. Allele coverage, mean pairwise genetic distance, mean expected heterozygosity (He), and mean PIC were calculated for the full collection and each optimized subset.

## 5. Conclusions

This study establishes a comprehensive molecular framework for evaluating elite popcorn inbred lines in Korea. Genome-wide SSR profiling revealed moderate but structured genetic diversity within a highly fixed parental pool shaped by repeated selfing and directional selection. Population structure analyses using STRUCTURE, PCoA, and UPGMA consistently identified clear genetic stratification and fine-scale hierarchical subdivision, reflecting historical recombination among related breeding backgrounds rather than strictly isolated heterotic groups. The complete homozygosity observed across SSR markers on chromosome 6 suggests a potential selection signal in genomic regions associated with popping-related traits. However, this observation requires further validation using high-density SNP genotyping or resequencing to confirm the presence of a potential selective sweep. Residual heterozygosity analysis further demonstrated generally acceptable fixation levels in commercial parental lines, although additional purification may enhance hybrid uniformity in certain cases. Beyond descriptive diversity analysis, this study also identified codominant SSR markers for F_1_ hybrid purity verification and implemented breeding-oriented subset optimization. The optimized representative panel retained full allelic coverage while increasing genetic divergence and reducing redundancy, demonstrating that operational efficiency can be improved without compromising genetic representation. Collectively, these findings advance our understanding of the genetic architecture of Korean popcorn germplasm and provide practical molecular resources for parental line selection, hybrid seed purity assurance, and precision breeding. By integrating genome-wide diversity assessment, fixation monitoring, hybrid purity marker development, population structure characterization, and subset optimization, this study establishes a strategic framework for molecularly informed popcorn breeding and germplasm management.

## Figures and Tables

**Table 1:** Genetic diversity parameters of 77 popcorn inbred lines estimated using 100 SSR markers.

Chr.		Na	Ne	I	Ho	He	MAF	PIC	F
1 (*n* = 12)	Mean	3.08	1.814	0.638	0.113	0.367	0.721	0.319	0.790
	Min	2	1.027	0.070	0.000	0.026	0.417	0.026	−0.553
	Max	5	2.976	1.165	0.789	0.664	0.987	0.594	1.000
2 (*n* = 9)	Mean	3.33	2.020	0.761	0.017	0.444	0.649	0.389	0.942
	Min	2	1.156	0.299	0.000	0.135	0.373	0.129	0.711
	Max	5	3.032	1.147	0.065	0.670	0.929	0.600	1.000
3 (*n* = 9)	Mean	2.67	1.874	0.646	0.050	0.407	0.664	0.335	0.834
	Min	2	1.054	0.138	0.000	0.051	0.312	0.050	0.179
	Max	4	3.523	1.303	0.263	0.716	0.974	0.661	1.000
4 (*n* = 9)	Mean	3.22	2.132	0.808	0.009	0.458	0.653	0.408	0.980
	Min	1	1.000	0.000	0.000	0.000	0.311	0.000	0.922
	Max	5	3.771	1.355	0.039	0.735	1.000	0.686	1.000
5 (*n* = 14)	Mean	2.93	1.954	0.707	0.020	0.444	0.631	0.365	0.894
	Min	2	1.013	0.040	0.000	0.013	0.434	0.013	−0.007
	Max	5	2.785	1.090	0.125	0.641	0.993	0.565	1.000
6 (*n* = 8)	Mean	2.63	1.440	0.458	0.000	0.265	0.820	0.227	1.000
	Min	2	1.054	0.122	0.000	0.051	0.574	0.050	1.000
	Max	4	1.958	0.865	0.000	0.489	0.974	0.421	1.000
7 (*n* = 10)	Mean	2.90	1.725	0.578	0.134	0.353	0.724	0.296	0.723
	Min	2	1.026	0.069	0.000	0.026	0.421	0.025	−0.387
	Max	5	2.893	1.081	0.681	0.654	0.987	0.581	1.000
8 (*n* = 9)	Mean	2.89	1.548	0.561	0.019	0.324	0.792	0.282	0.926
	Min	2	1.054	0.122	0.000	0.051	0.638	0.050	0.686
	Max	6	2.177	1.051	0.052	0.541	0.974	0.497	1.000
9 (*n* = 10)	Mean	2.80	1.651	0.573	0.012	0.340	0.756	0.290	0.942
	Min	2	1.048	0.111	0.000	0.046	0.506	0.045	0.659
	Max	5	2.517	0.996	0.026	0.603	0.977	0.525	1.000
10 (*n* = 10)	Mean	3.40	1.788	0.582	0.134	0.296	0.785	0.274	0.787
	Min	2	1.026	0.069	0.000	0.026	0.286	0.025	−0.207
	Max	7	4.661	1.666	0.948	0.785	0.987	0.753	1.000
	Total	2.99	1.805	0.635	0.053	0.373	0.715	0.321	0.875

Na, mean number of alleles per locus; Ne, effective number of alleles; I, Shannon’s diversity index; Ho, observed heterozygosity; He, expected heterozygosity; MAF, major allele frequency; PIC, polymorphism information content; F, fixation index (inbreeding coefficient).

**Table 2:** Residual heterozygosity of the seven parental popcorn inbred lines and selected lines with the lowest and highest values based on 100 SSR markers.

Inbred Line	Missing	Valid	Heterozygous	Residual Heterozygosity (%)
GP1	1	99	4	4.04
GP2	6	94	7	7.45
GP3	0	100	4	4.00
GP4	6	94	5	5.32
GP5	2	98	4	4.08
GP6	1	99	6	6.06
GP7	2	98	7	7.14
21PS8142	0	100	2	2.00
21PS8113	4	96	2	2.08
20PS8102	5	95	2	2.11
21PS8001	8	92	2	2.17
21PS8144	0	100	3	3.00
20PS8064	0	100	3	3.00
20PS8118	4	96	11	11.46
21PS8043	1	99	12	12.12

**Table 3:** Polymorphic SSR markers distinguishing parental lines for purity testing in five F_1_ popcorn hybrids.

F_1_ Hybrid Varieties	Polymorphic Markers Between Two Parental Lines
Oryun Popcorn (GP1 × GP2)	umc2309, umc2224, umc1871, umc2293, umc2301, umc2043, umc1098, bnlg1036, umc1893, umc2137, umc2140, umc1853, umc1865, umc1314, umc2026, umc2373, bnlg1176, umc1192, umc1401, umc1051, umc1420, bnlg1138
G-Popcorn (GP3 × GP4)	umc1887, umc2309, umc2199, umc1871, umc2293, umc2215, umc2301, phi101049, umc1043, umc2043, umc1098, umc1715, umc2240, bnlg1036, umc2396, umc1973, umc2140, umc1853, umc1978, umc1314, umc1462, umc2219, umc1743, umc2373, umc1707, bnlg1525, umc2362, umc1401, umc2053, umc1066, umc1632, umc1051, umc1790, umc1101
Oryun Popcorn 2 (GP5 × GP3)	umc1814, umc2309, umc1934, umc2101, umc2081, umc1715, umc1853, umc2367, umc1865, umc1846, umc2219, umc1667, bnlg1176, umc1790, bnlg1138, umc1101
Kichan Popcorn (GP6 × GP3)	umc2309, umc1934, umc2101, umc2199, phi374118, umc2293, umc2301, umc1823, phi101049, umc2081, umc1043, umc1715, umc2294, bnlg1036, umc1598, umc1973, umc2137, umc2140, umc1865, umc1314, umc2219, umc1557, umc1447, bnlg1518, umc1519, umc1844, bnlg1525, umc1192, umc1401, umc1051, umc1790, umc2275, umc1420, bnlg1138, umc1101
Alchan Popcorn (GP2 × GP7)	umc2309, umc1934, umc2101, phi374118, umc2293, umc2301, phi101049, umc2081, umc1043, umc1098, umc1715, umc2240, bnlg1036, bnlg1662, umc1973, umc2137, umc2140, umc1865, umc1846, umc1314, umc1743, umc2172, umc1432, umc2373, bnlg1525, umc2053, umc2351, umc1051, umc2275, bnlg1138

**Table 4:** Popcorn inbred lines developed at the Maize Experimental Station in South Korea.

Entry	Accession	STRUCTURE (*K* = 2)	UPGMA	PCoA	Entry	Accession	STRUCTURE (*K* = 2)	UPGMA	PCoA
1	21PS8001	Group I	1-II	1	40	23PS8013	Admixed	11	4
2	21PS8010	Group I	1-II	1	41	23PS8019	Admixed	6	4
3	21PS8012	Group I	1-III	4	42	23PS8020	Admixed	1-V	4
4	21PS8017	Group I	1-II	1	43	23PS8026	Admixed	6	3
5	21PS8020	Group I	1-II	1	44	23PS8033	Group I	1-III	4
6	21PS8024	Group I	1-II	1	45	GP2	Admixed	1-X	1
7	21PS8041	Admixed	1-VI	4	46	GP1	Group I	1-II	1
8	21PS8043	Admixed	1-IV	4	47	17PS8019	Admixed	1-II	1
9	21PS8058	Admixed	1-IV	3	48	GP3	Group II	1-I	2
10	21PS8076	Group I	1-II	1	49	GP4	Group II	2	3
11	21PS8081	Group I	1-II	1	50	GP5	Group I	1-II	1
12	21PS8085	Admixed	1-I	2	51	GP6	Group I	1-III	4
13	21PS8086	Admixed	1-IX	4	52	GP7	Admixed	7	2
14	21PS8087	Group II	1-I	2	53	20PS8001	Admixed	1-III	4
15	21PS8090	Admixed	4	1	54	20PS8004	Admixed	2	1
16	21PS8093	Group II	1-I	3	55	20PS8009	Admixed	7	1
17	21PS8094	Admixed	1-I	2	56	20PS8012	Group II	1-VII	3
18	21PS8101	Admixed	9	3	57	20PS8015	Group II	1-VII	3
19	21PS8106	Group II	1-I	2	58	20PS8018	Admixed	1-VIII	2
20	21PS8111	Group II	1-I	2	59	20PS8020	Admixed	1-VI	4
21	21PS8113	Group II	1-I	2	60	20PS8022	Group II	2	2
22	21PS8119	Group II	1-I	2	61	20PS8024	Group II	2	2
23	21PS8138	Group II	1-I	2	62	20PS8026	Group II	2	2
24	21PS8142	Admixed	10	2	63	20PS8041	Admixed	1-II	1
25	21PS8144	Group II	1-I	2	64	20PS8048	Admixed	1-II	2
26	22PS8001	Group I	1-III	4	65	20PS8053	Group I	1-III	4
27	22PS8004	Group I	1-II	1	66	20PS8059	Group I	1-II	1
28	22PS8006	Admixed	3	3	67	20PS8064	Admixed	1-III	4
29	22PS8008	Admixed	3	3	68	20PS8067	Group I	1-III	4
30	22PS8010	Admixed	3	3	69	20PS8077	Group II	1-I	2
31	22PS8012	Admixed	3	3	70	20PS8084	Group II	1-I	3
32	22PS8014	Admixed	5	3	71	20PS8097	Admixed	1-I	3
33	22PS8018	Admixed	5	3	72	20PS8102	Admixed	4	1
34	22PS8020	Group II	1-I	2	73	20PS8109	Admixed	12	1
35	22PS8022	Group II	1-I	2	74	20PS8112	Admixed	8	1
36	23PS8003	Group I	1-V	4	75	20PS8114	Admixed	8	4
37	23PS8006	Group II	1-I	2	76	20PS8116	Admixed	1-IV	4
38	23PS8009	Admixed	1-V	4	77	20PS8118	Admixed	1-IV	4
39	23PS8011	Group I	1-V	4					

**Table 5:** Comparison of three optimized representative panels relative to the full popcorn inbred collection.

Set	Lines (n)	Allele Coverage (%)	Mean Pairwise Distance	Mean He	Mean PIC
Entire set (77)	77	100	0.393	0.373	0.321
PowerCore (36)	36	100	0.430	0.396	0.341
CoreHunter (36)	36	100	0.450	0.414	0.357
Intersection (27)	27	100	0.456	0.415	0.357

Allele coverage (%) represents the proportion of total alleles retained in each subset relative to the entire set of 77 inbred lines.

## Data Availability

All data generated or analyzed during this study are included in this published article and its Supplementary Materials.

## Supplementary Materials

The following supporting information can be downloaded at: https://www.mdpi.com/article/10.3390/plants15152324/s1, Table S1. Genetic diversity statistics, chromosomal locations, and primer sequences of 100 SSR loci evaluated in 77 popcorn inbred lines; Table S2. Residual heterozygosity of 77 popcorn inbred lines estimated using 100 SSR markers; Table S3. Pedigree information and breeding backgrounds of the 77 popcorn inbred lines used in this study; Figure S1. Magnitude of ∆*K* as a function of *K*. The peak value of ∆*K* was observed at *K* = 2, suggesting two genetic clusters among the 77 popcorn inbred lines.
