# Supplementary material for: Genetic Diversity, Population Structure and Parental Panel Optimization in Elite Popcorn (Zea mays L.) Inbred Lines Revealed by SSR Markers
Source: Plants (Basel). 2026 Jul 28;15(15):2324. doi: 10.3390/plants15152324 (PMC13468367; doi:10.3390/plants15152324)
Supplement: Supplementary file 1 [file plants-15-02324-s001.zip › Supplementay Figure S1.pdf]

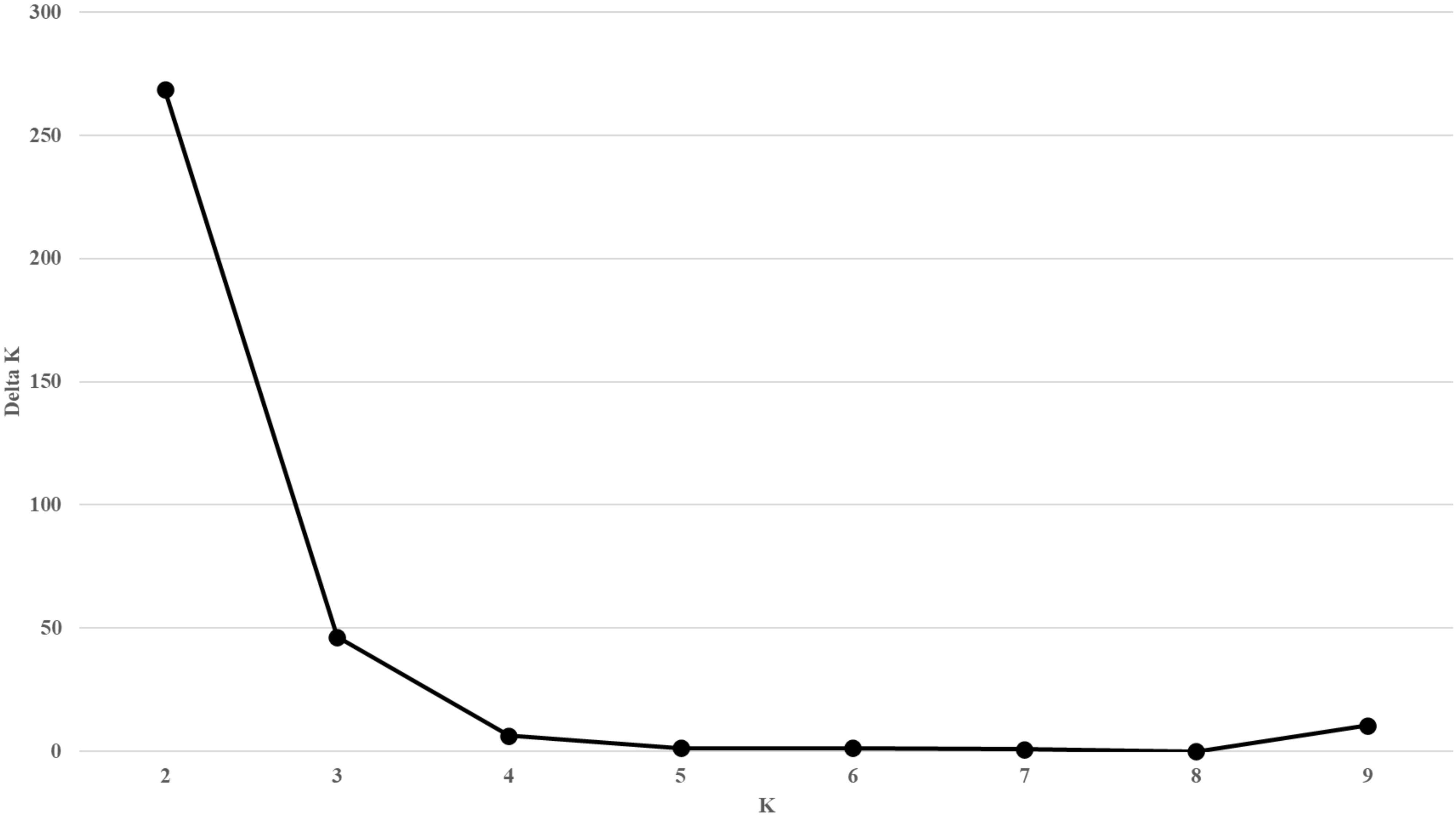

Figure S1. Magnitude of Δ*K* as a function of *K*. The peak value of Δ*K* was observed at *K*=2, suggesting two genetic clusters among the 77 popcorn inbred lines.
