# Supplementary material for: Genetic Diversity, Population Structure and Parental Panel Optimization in Elite Popcorn (Zea mays L.) Inbred Lines Revealed by SSR Markers
Source: Plants (Basel). 2026 Jul 28;15(15):2324. doi: 10.3390/plants15152324 (PMC13468367; doi:10.3390/plants15152324)
Supplement: Supplementary file 1 [file plants-15-02324-s001.zip › Supplementay Table S1.pdf]

Table S1. Genetic diversity statistics, chromosomal locations, and primer sequences of 100 SSR loci evaluated in 77 popcorn inbred lines.

Marker	Chr.	Forward Sequence	Reverse Sequence	Na	Ne	I	Ho	He	MAF	PIC	F
umc2215	1.01	ATGCATGATGACGACATGATAAGC	CATGGCACCATACATGTAAGCAC	3	1.800	0.748	0.000	0.445	0.701	0.382	1.000
umc1177	1.01	CGTGTACCGCTCCTCTATAGTCGT	AAGTGGCCGAATTCATCCTTTATT	3	1.111	0.233	0.000	0.100	0.948	0.096	1.000
umc1269	1.01	TATATTAGAGGCACCTCCCTCCGT	AGCTGCTTCAGCGACTTTGG	3	2.034	0.779	0.789	0.508	0.579	0.403	-0.553
umc2224	1.01	CGGGCTATAAGTAAACTGAAACGG	AGCTGGCTAGCTGTTAGCGTTTT	5	2.201	1.067	0.013	0.546	0.638	0.506	0.976
umc1598	1.03	CACGTAATAATCCTTTTCCCCAGA	TTTTCTGCAAATGCTCAGAATGAT	4	2.789	1.165	0.493	0.641	0.486	0.580	0.231
umc2396	1.06	TGCATGCATTTTTAGGTTTGGAAT	TGCATCTTTAGCTACGAGACAACCT	4	2.294	0.954	0.000	0.564	0.545	0.480	1.000
umc1715	1.07	TTCATTGGGTCTCTAGCCAAGAAG	GGGGAGTCACAGATCTCATCAACT	4	2.976	1.139	0.000	0.664	0.417	0.594	1.000
umc2240	1.08	CGCCTTTGTAACCCAGACTCATTA	CGGATGTTGCCAAGTACATCATATC	2	1.913	0.670	0.013	0.477	0.607	0.363	0.972
umc1013	1.08	TAATGTGTCCATACGGTGGTGG	AGCTGGCTAGTCTCAGGCACTC	2	1.055	0.123	0.000	0.052	0.973	0.051	1.000
umc1118	1.11	ATCAGATTCCGAAGGGTCCATAAT	GTAGTGAAATGAATCGTGAGAGCG	2	1.027	0.070	0.000	0.026	0.987	0.026	1.000
umc2241	1.11	CGTGATCGACATGGACCACTT	CACCATCACATTCACACGCATAG	2	1.027	0.071	0.000	0.026	0.987	0.026	1.000
umc1725	1.12	CTACGGCCAGAGTATCGGTCAC	TATGGTGGGAGAGACGACAGGTAT	3	1.547	0.638	0.052	0.354	0.786	0.318	0.853
umc1227	2.01	CAAGTTGGTGAGATGGATCTGTTG	GCTCCTGGGTCTTCCTCTCC	3	1.156	0.299	0.039	0.135	0.929	0.129	0.711
umc1823	2.02	AAAGCCTTACTGTTATTAGGCTAGGCA	AGAAAACCAGCCCCAGATGTTC	5	2.419	1.041	0.000	0.587	0.506	0.505	1.000
umc1934	2.02	TCCGGGACTAGTTGCTTGGTAGTA	TAGTGGTATCGTGATTCATGGGC	4	2.204	0.879	0.065	0.546	0.506	0.444	0.881
umc1946	2.06	GAAACGACCAGCACAGCACAT	GCACCACACCATCAGATCCAG	2	1.339	0.420	0.000	0.253	0.851	0.221	1.000
bnlg1036	2.06	GGGAGTATGGTAGGGAACCC	AAACCCTTGGAGCATACCCT	4	3.032	1.147	0.013	0.670	0.373	0.600	0.980
bnlg1138	2.06	TGCTCTAGCCGACCTCAATT	ATGCCTGAACCGTGATTAGG	3	2.733	1.051	0.014	0.634	0.473	0.561	0.979
umc1560	2.07	CGTTCGTCTCTGGGTAGCGTAG	TATAACAGCCTGCTGCTGCTTG	2	1.341	0.422	0.013	0.254	0.851	0.222	0.949
bnlg1662	2.08	GCACCCACATGAAGTATCCC	TTGTTTTTGCAGTGCCTCAG	4	1.484	0.592	0.000	0.326	0.803	0.288	1.000
phi101049	2.10	CCGGGAACTTGTTCATCG	CCACGTCCATGATCACACC	3	2.469	0.998	0.013	0.595	0.552	0.529	0.978
umc2101	3.00	CCCGGCTAGAGCTATAAAGCAAGT	CTAGCTAGTTTGGTGCGTGGTGAT	4	3.523	1.303	0.000	0.716	0.312	0.661	1.000
phi374118	3.02	TACCCGGACATGGTTGAGC	TGAAGGGTGTCCTTCCGAT	2	1.984	0.689	0.026	0.496	0.545	0.373	0.948
umc1814	3.02	AGAAGAAGAGGAGGTTCCATGACC	GCATGTTTCCCTTTCACCTCC	2	1.592	0.559	0.000	0.372	0.753	0.303	1.000

umc1973	3.05	CAGGCAGAAAAGGAACGGAAC	GTGCGAGAGAAGATGGATGATTG	2	1.610	0.566	0.000	0.379	0.746	0.307	1.000
umc1949	3.06	TGTAAACAGGTTGATCGTTGGAGA	TGTTTTGATCGCTTCACGCA	3	1.054	0.138	0.000	0.051	0.974	0.050	1.000
umc2269	3.06	GATGTCCATCTTGAAGACCACCAG	ATCCCTCCACCCTCCTCGTC	3	1.242	0.399	0.160	0.195	0.893	0.183	0.179
umc2275	3.07	CTCAAACTGCTCCACACTAGATGC	GTAATGCATGCACCAAGAAACAAT	3	2.035	0.750	0.000	0.509	0.539	0.392	1.000
umc2081	3.08	TTTTTCCACTTGGCTGCTGG	CAGTTTTTCAGAAACGATTTGCCT	3	2.096	0.793	0.000	0.523	0.519	0.411	1.000
umc1844	3.08	GGCATGGGTCTCTCATAAAGTCAT	CGACGTATATGGCTGAGAACCCTA	2	1.730	0.613	0.263	0.422	0.697	0.333	0.377
umc1043	4.07	CTGCGCATGAGAAAACAGTGG	TCCATCTCTGGCTGGAGGTTAAT	4	2.020	0.945	0.013	0.505	0.669	0.461	0.974
umc1871	4.08	CATGCAAGCTTATCCTCCTCACTT	CTCTTTTCCTGCCTGCTAGAGATG	5	2.975	1.233	0.000	0.664	0.468	0.606	1.000
umc2137	4.08	ACCACTGCAACCTAGAGCTGTACC	CACTTGGGTTGTTCCACAGGAG	3	2.387	0.978	0.014	0.581	0.570	0.516	0.976
umc1667	4.08	AAACATACCCCACCACTGCAAG	CGAAGAACATGTAACACAGCAACC	3	1.925	0.789	0.000	0.481	0.658	0.405	1.000
umc1051	4.08	CTGATCTGACTAAGGCCATCAAAC	AATGATCGAAATGCCATTATTTGT	4	3.771	1.355	0.000	0.735	0.311	0.686	1.000
umc1101	4.09	GCTGAAAAACGGAGTTCATATGGT	AAGCTTATCCACCTCGAGGAAAAC	3	1.995	0.780	0.039	0.499	0.610	0.403	0.922
umc1197	4.11	GGTGTAATTTAGGGAGTGTTTGTTCG	CCGCATAGATGTGCTTTCTAGGAG	1	1.000	0.000	0.000	0.000	1.000	0.000	-
umc1707	4.11	GTCGAGAAGCGATCGATCATAGTAG	GCACGACAGGGAGTACGGTC	3	1.887	0.817	0.013	0.470	0.691	0.419	0.972
umc2290	4.11	GAATCACAATGGATCCTACCAAGG	AACTGCAAGCAAAATCAACACAAG	3	1.227	0.370	0.000	0.185	0.899	0.172	1.000
umc2293	5.03	ATGTTCCGTTTATTATTTGCCCG	AAAGAACAGACGCGATCCAATC	4	2.160	0.856	0.000	0.537	0.500	0.430	1.000
umc2294	5.03	CCCACCATTCTATATATTGTTGCCA	ATTGGAGTGGCTCCATTGCTT	2	1.907	0.669	0.000	0.476	0.610	0.363	1.000
umc2140	5.03	CTAGGGTTTGCCTCTTCCCTCTC	AGTCTAGAGGAAGTCGGAATTGGG	2	1.876	0.660	0.014	0.467	0.628	0.358	0.971
umc2159	5.03	GTTATTTCTGAACCATGGGACTGTG	CTGTATGGGCTCGTATTGTCACCT	3	1.058	0.148	0.000	0.055	0.972	0.054	1.000
umc1557	5.03	CTAGTTCTCTCAAAAGCGCCG	ACAATAGGTAGGTCCCCTGCTTT	2	1.939	0.677	0.000	0.484	0.589	0.367	1.000
umc1447	5.03	TAATACTCCTACTAACGGCGCTGC	TCTGTCTCCCATGCCTGAAATAAT	3	2.078	0.826	0.125	0.519	0.590	0.426	0.759
umc1468	5.03	CGAAAAGCCCTAGTGCTCCG	TTTCTGTAAAAGGGATCTGGTGGG	2	1.013	0.040	0.013	0.013	0.993	0.013	-0.007
umc2301	5.04	AATGCGTTGTGCTGTGAAATG	CTAGAAGCTACAGCGAGTGAGGAC	4	2.573	1.041	0.027	0.611	0.452	0.531	0.955
umc1098	5.04	ATAACTCCTGCATCTTGTCAGCAA	CACAGGATAGAACCTTCTCAGCAAA	5	2.620	1.090	0.026	0.618	0.442	0.541	0.958
umc1224	5.04	CTGAGAGGTCCCAAAGGAGTACAA	ATGACCTGCACACAGAAAGAACAA	2	1.525	0.528	0.000	0.344	0.779	0.285	1.000
umc2373	5.04	ACCCAAGTGAGGTGAAGTGAAGC	TATGGTACAGGCACAGCAGCAGTA	4	1.832	0.851	0.000	0.454	0.711	0.413	1.000

umc1192	5.04	GATCGAACTGCAGAGGAACAAGAG	CGCTCCACATCCACATCTACAC	2	1.885	0.662	0.000	0.470	0.623	0.359	1.000
umc1853	5.05	TTATTATTAACACCTGCCTGCGCT	GCTAGCTAGGAAACATGGCTTGTC	3	2.785	1.058	0.079	0.641	0.434	0.565	0.877
umc2026	5.05	TTCGACATAGCAGACGAAACTCTG	ACTGCACCCTAGTGTGATGTTCCT	3	2.104	0.797	0.000	0.525	0.507	0.412	1.000
umc2309	6.00	GTCGAAATTCCTGACACAAAAAGG	CTGTGTTTTGTGTATTAGCGCCAG	4	1.882	0.865	0.000	0.469	0.694	0.421	1.000
umc1887	6.03	CTTGCCATTTTAATTTGGACGTTT	CGAAGTTGCCCAAATAGCTACAGT	3	1.810	0.714	0.000	0.447	0.684	0.368	1.000
umc1314	6.05	ACAAACTAAACTGCATGTACCCCC	CACAAATGATGTGGTGGCAATATC	2	1.958	0.682	0.000	0.489	0.574	0.370	1.000
umc1341	6.05	GTCTACCAGGACGTTTACCTGTGG	CCTCAATCCTTTGTGGACAAACAC	3	1.227	0.370	0.000	0.185	0.899	0.172	1.000
umc1462	6.06	GAACAGCAAGCCAACAAATAATCA	GCTGCATTGCATTACATGATAGGA	2	1.084	0.170	0.000	0.078	0.959	0.075	1.000
umc1762	6.06	CTTACTCCAGGCACTCCATACCAT	ATCCAGGTGAATGGTGTTTACGAT	3	1.301	0.432	0.000	0.232	0.868	0.210	1.000
umc1350	6.07	GACAGAAGAGGATCAGCCGAAAT	GAGATCTACGGGAAGCCCACTT	2	1.201	0.307	0.000	0.167	0.908	0.153	1.000
phi364545	6.07	TAAGCAAAGCAAGGCAACC	TCGCCTCACTCTCACACTCC	2	1.054	0.122	0.000	0.051	0.974	0.050	1.000
umc1066	7.01	ATGGAGCACGTCATCTCAATGG	AGCAGCAGCAACGTCTATGACACT	4	1.748	0.710	0.013	0.428	0.708	0.356	0.970
umc1632	7.01	GGACACTTCCTTCCACGAAAAA	CGCAGATGGATGAATAACCTCTTC	2	1.414	0.468	0.000	0.293	0.822	0.250	1.000
umc1068	7.02	AGTCGTTTTCAAAGGCTGCTGATA	TGAGTCACCTCATTTCTTCTGGTTC	2	1.040	0.096	0.013	0.038	0.981	0.037	0.660
umc2326	7.02	CGATGATGGGTGTTGTGGTTAGAT	GGTGAGATACAGAGGTGGATGAGG	2	1.026	0.069	0.000	0.026	0.987	0.025	1.000
umc1978	7.02	CACTCCCTCCATTTCTCTCACCT	ATCGCTACCATTGGACGCTTTAC	2	1.885	0.662	0.052	0.470	0.623	0.359	0.889
umc2092	7.02	GCACAACCTACTCGCTACCTTACC	ATCGTACTACTCCACTTGCTTGGC	3	2.893	1.081	0.539	0.654	0.421	0.581	0.176
umc1401	7.02	CTCTGGTCCATCCTCATCGACT	TCTCTTGATCACATATCGATCCCA	3	2.292	0.901	0.026	0.564	0.468	0.466	0.954
umc1865	7.03	GAGGAGTTAGGGTCCGTCCATAC	CTCTTCCTCTCCGTCTCGCTC	3	1.880	0.736	0.013	0.468	0.656	0.380	0.972
umc1275	7.03	GTTCGCAGGCCTAGACTCTAATGA	AGTTCTAGTCCCTGGTGATCCTCC	3	1.112	0.243	0.000	0.101	0.947	0.099	1.000
umc1242	7.05	TGATCCAAGAAAAGCAAAGCAAAC	TGGCAGTCTGTATAGTTGTCCGAA	5	1.965	0.815	0.681	0.491	0.630	0.402	-0.387
umc1790	8.02	GAGAGGGAGGAGCAAGGAAATAGA	GAGAAGAGTTCTCCATGGAACGAG	4	1.757	0.761	0.013	0.431	0.717	0.375	0.969
umc2199	8.05	ACCGAGTTAAGATTACATCACGCC	TGTTTCCCCTAATAAAGCAAATGAA	2	1.317	0.405	0.013	0.241	0.860	0.212	0.945
umc2210	8.05	AGCGGGTCGATCTTTCTCTTAGTT	GATGCTACCATTTCAGTGAGCGAT	2	1.054	0.122	0.000	0.051	0.974	0.050	1.000
umc1712	8.05	TACCTACCTTTTGATGCAGGTTGC	CGGCACTAGTGAGTCTGATCAATG	2	1.198	0.305	0.052	0.165	0.909	0.152	0.686
umc2367	8.05	AACCGTTGTCGTTGTCTCTGTTC	ATGAACTTGGTCACGCTCATCTC	2	1.736	0.615	0.013	0.424	0.695	0.334	0.969

umc1846	8.05	ATTATTGGTCACAGGCCCTACCTT	TTAGGCCCTCGTCTTGTAGACTTG	4	1.781	0.811	0.000	0.439	0.722	0.397	1.000
bnlg1176	8.05	ACTCCTCAAAACCTAGGTGACA	CACCGATGATGGTGAGTACG	6	2.177	1.051	0.013	0.541	0.638	0.497	0.976
umc2037	8.06	TTCGCATTACTACGTTTGGTTCCT	CCCACAGGACTGGTGTATCTGA	2	1.396	0.458	0.026	0.284	0.829	0.243	0.907
umc2395	8.06	ATCACATCTTGCGTTGTCATTTTG	ATGGATTCTTTCCGGCCTCTC	2	1.515	0.523	0.039	0.340	0.783	0.282	0.884
umc2362	9.01	TTTCTGAAACGCAACTATCCACG	TGACCAGTGTTCATGACCTCATCT	3	2.517	0.996	0.026	0.603	0.506	0.525	0.957
umc1893	9.02	TCCAGTGCCACCCCTAGATAGTAA	ACCCCAGAGTATCTCATCACCCTT	3	1.950	0.792	0.014	0.487	0.646	0.407	0.971
umc2213	9.02	TGATTTGATCCATTACCATCCTATTC	TGCGTGTGTCTGTCTGTCTGTTAT	2	1.048	0.111	0.016	0.046	0.977	0.045	0.659
umc2219	9.02	TGAGCTCGTACAAATGCTGTGATT	TTCTTTTCACTTGTCAAACATGGG	2	1.811	0.640	0.000	0.448	0.662	0.348	1.000
phi027	9.03	CACAGCACGTTGCGGATTTCTCT	GCGTACGTACGACGAAGACAC	3	1.112	0.243	0.000	0.101	0.947	0.099	1.000
umc1743	9.03	TGGACTTCGAAAATTCTCTTCAGC	GAGAGGAGGAGCTTCACGAGC	2	1.525	0.528	0.026	0.344	0.779	0.285	0.925
umc1420	9.03	AAGACGACCTCGTTTGTAGCCTC	GTATTTCAACAGCTGGGACGGT	2	1.659	0.587	0.013	0.397	0.727	0.318	0.966
umc1519	9.04	CTCGAGACTCTGGTTCAATCCAAT	CATGCACGTACTTCCCTGATTTTT	5	1.485	0.683	0.013	0.327	0.812	0.307	0.960
umc2338	9.05	ATCTCACACTCACGACCTGTTGTC	AGCATTGGGACTGTTCAAGAGAAG	2	1.081	0.165	0.000	0.075	0.961	0.072	1.000
bnlg1525	9.07	AGGAATTGCGAGTCTTCCAA	CAACCCCCAAAATGAACAAA	4	2.324	0.984	0.013	0.570	0.546	0.489	0.977
umc2053	10.01	ATCTCTCCCTCGCTCTCCTTCTC	AGCAGCAGGTTGGTCGAATG	2	1.138	0.240	0.000	0.121	0.935	0.114	1.000
umc1432	10.02	GGCCATGATACAGCAAGAAATGAT	TACTAGATGATGACTGACCCAGCG	3	1.380	0.540	0.013	0.275	0.844	0.258	0.953
umc1239	10.03	ATCAACACACCTTTCGATTTCTGG	CGGTGATTAGTCGATGAAGAGTGA	2	1.513	0.522	0.081	0.339	0.784	0.281	0.761
bnlg1518	10.04	AGCTGTACACGCAGTAGGCA	GGCTCTGTTAATTCGATCGC	4	1.275	0.464	0.000	0.216	0.882	0.205	1.000
umc1330	10.04	AGCAAAGAAGCCAAAGAGCAACT	GTCACCACCGTCTGCTGGTA	2	1.026	0.069	0.000	0.026	0.987	0.025	1.000
umc1995	10.04	AAACAATTGTCATCTTCTTCTGCG	GTCAGACCGACGCACTCACTAA	7	4.661	1.666	0.948	0.785	0.286	0.753	-0.207
umc2043	10.05	GAGGCATACGGCATACCATACC	GTAGGAGAAACAGGTGCTGGTGTC	4	2.953	1.189	0.013	0.661	0.461	0.601	0.980
umc2172	10.07	AGTAATCTCATGCCCGCTCTTCTT	TACGCTGAGTCAAAAACCAGGAAT	5	1.850	0.916	0.286	0.460	0.714	0.428	0.378
umc1645	10.07	CATCGTCATCAGTTCTTCCATGAG	GTGATTGACAGAGGACCATCAGAA	2	1.031	0.079	0.000	0.030	0.985	0.029	1.000
umc2351	10.07	CGGGCAGACAGACATTATTTCAA	CTGGCTGTCTGTCGTCGTCTCT	3	1.054	0.138	0.000	0.051	0.974	0.050	1.000

Chromosome and bin positions (chr.) correspond to physical map locations in maize. Na, number of alleles; Ne, effective number of alleles; I, Shannon's information index; Ho, observed heterozygosity; He, expected heterozygosity; MAF, major allele frequency; PIC, polymorphism information content; F, fixation index.
