# Supplementary material for: Genetic Diversity, Population Structure and Parental Panel Optimization in Elite Popcorn (Zea mays L.) Inbred Lines Revealed by SSR Markers
Source: Plants (Basel). 2026 Jul 28;15(15):2324. doi: 10.3390/plants15152324 (PMC13468367; doi:10.3390/plants15152324)
Supplement: Supplementary file 1 [file plants-15-02324-s001.zip › Supplementay Table S3.pdf]

Table S3. Pedigree information and breeding backgrounds of the 77 popcorn inbred lines used in this study.

Entry	Accession	Pedigree	Entry	Accession	Pedigree
1	21PS8001	13popS003-C2-1-2-1-1-2-1-1	40	23PS8013	15PS0002-2-1-2-1-1-1-1
2	21PS8010	13popS003-C2-3-3-1-2-2-1-1	41	23PS8019	15PS0003-1-2-1-1-1-1-1
3	21PS8012	13popS003-C4-3-3-2-1-1-1-1	42	23PS8020	15PS0003-1-2-1-1-2-1-1
4	21PS8017	13popS006-1-2-2-1-1-3-1-1	43	23PS8026	15PS0003-1-2-1-2-1-1-1
5	21PS8020	13popS006-1-2-2-1-2-1-1-1	44	23PS8033	15PS0003-1-2-1-2-2-1-3
6	21PS8024	13popS006-1-2-2-1-2-2-1-1	45	GP2	9103-5-2-2-2-1-1-1-1-1
7	21PS8041	13popS015-C1-1-1-1-1-2-1-1	46	GP1	9101-4-5-1-3-1-2-1-1-1
8	21PS8043	13popS016-2-1-3-1-2-1-1-1	47	17PS8019	09PS0022-1-2-1-1-5-2-1-1
9	21PS8058	13popS017-2-3-3-1-1-2-1-1	48	GP3	9108-1-3-1-2-1-1-1-1-1
10	21PS8076	13popS020-C1-2-1-2-1-1-1-1	49	GP4	9106-4-2-5-1-1-1-T1-1-1-1
11	21PS8081	13popS020-C3-3-3-1-2-1-1-1	50	GP5	9103-1-1-2-2-1-1-1-1-1
12	21PS8085	13popS022-1-2-3-1-2-2-1-1	51	GP6	9104-1-7-1-2-1-1-1-1-1
13	21PS8086	13popS024-C1-3-1-1-3-1-1-1	52	GP7	080004-1-1-2-1-1-2-1-1-1
14	21PS8087	13popS030-C2-2-2-2-1-1-1-1	53	20PS8001	12popfemale-2-C2-4-1-2-1-1-1
15	21PS8090	13popS033-C4-1-2-2-1-1-1-1	54	20PS8004	12popfemale-4-2-2-1-1-1-1-1
16	21PS8093	13popS034-1-2-1-1-1-2-1-1	55	20PS8009	12popfemale-18-1-2-2-1-1-1-1
17	21PS8094	13popS037-2-2-1-1-1-1-1-1	56	20PS8012	12popfemale-18-1-2-2-2-1-2-1
18	21PS8101	13popS038-C2-2-1-1-1-2-1-1	57	20PS8015	12popfemale-18-1-2-2-2-2-1-1
19	21PS8106	13popS040-C2-1-2-1-1-2-1-1	58	20PS8018	12popfemale-25-C1-2-1-1-1-1-1
20	21PS8111	13popS040-C2-1-3-2-1-1-1-1	59	20PS8020	12popfemale-25-C2-1-1-1-1-1-1
21	21PS8113	13popS040-C2-3-1-1-1-1-1-1	60	20PS8022	12popfemale-36-2-3-1-1-2-2-1
22	21PS8119	13popS040-C3-3-1-1-1-1-1-1	61	20PS8024	12popfemale-36-2-3-1-1-3-2-1
23	21PS8138	13popS040-C3-3-3-1-2-1-1-1	62	20PS8026	12popfemale-36-2-3-3-1-1-1-1
24	21PS8142	13popS044-C1-2-1-1-1-1-1-2	63	20PS8041	12PS0083-1-2-2-2-2-1-1-1
25	21PS8144	13popS044-C1-2-2-1-1-1-1-2	64	20PS8048	12PS0084-1-1-4-1-1-2-2-1
26	22PS8001	14popS0001-1-1-1-1-1-1-1-1	65	20PS8053	12PS0086-1-1-1-2-1-1-1-1
27	22PS8004	14popS0001-1-2-1-1-1-1-3-1	66	20PS8059	12PS0089-1-C4-2-1-1-1-2-1
28	22PS8006	14popS0002-1-2-1-1-2-1-2-1	67	20PS8064	12PS0106-1-3-1-2-1-1-1-1
29	22PS8008	14popS0002-1-2-1-1-2-1-3-1	68	20PS8067	12PS0107-1-1-2-1-1-1-1-1
30	22PS8010	14popS0002-1-2-1-1-3-1-2-1	69	20PS8077	12PS0111-1-4-1-1-1-2-1-1
31	22PS8012	14popS0002-3-1-1-2-2-1-1-1	70	20PS8084	12PS0113-1-1-3-1-1-1-1-1
32	22PS8014	14popS0005-2-2-1-2-1-1-1-1	71	20PS8097	12PS0118-1-3-2-1-4-1-1-1
33	22PS8018	14popS0005-2-2-1-2-1-1-2-1	72	20PS8102	12PS0123-1-1-2-2-1-1-1-2
34	22PS8020	14popS0006-1-2-1-1-1-1-1-1	73	20PS8109	12PS0146-1-1-3-2-1-2-1-1
35	22PS8022	14popS0006-1-2-1-1-1-1-1-3	74	20PS8112	12PS0155-1-C1-2-3-1-1-2-1
36	23PS8003	15PS0001-1-1-1-2-1-2-1-1	75	20PS8114	12PS0155-1-C1-2-3-3-1-1-1
37	23PS8006	15PS0001-1-1-1-2-1-2-2-1	76	20PS8116	12PS0156-1-C2-5-2-2-1-1-1
38	23PS8009	15PS0001-1-1-1-2-2-2-1-1	77	20PS8118	12PS0156-1-C2-2-2-1-1-1-1
39	23PS8011	15PS0001-1-1-1-2-2-2-2-1			
