# Supplementary material for: Genetic Diversity, Population Structure and Parental Panel Optimization in Elite Popcorn (Zea mays L.) Inbred Lines Revealed by SSR Markers
Source: Plants (Basel). 2026 Jul 28;15(15):2324. doi: 10.3390/plants15152324 (PMC13468367; doi:10.3390/plants15152324)
Supplement: Supplementary file 1 [file plants-15-02324-s001.zip › Supplementay Table S2.pdf]

Table S2. Residual heterozygosity of 77 popcorn inbred lines estimated using 100 SSR markers.

Inbred line	Missing	Valid	Heterozygous	Residual heterozygosity (%)	Inbred line	Missing	Valid	Heterozygous	Residual heterozygosity (%)
21PS8142	0	100	2	2.00	20PS8024	3	97	5	5.15
21PS8113	4	96	2	2.08	21PS8076	4	96	5	5.21
20PS8102	5	95	2	2.11	20PS8109	4	96	5	5.21
21PS8001	8	92	2	2.17	22PS8014	5	95	5	5.26
21PS8144	0	100	3	3.00	GP4	6	94	5	5.32
20PS8064	0	100	3	3.00	22PS8018	7	93	5	5.38
21PS8093	1	99	3	3.03	20PS8112	7	93	5	5.38
21PS8119	1	99	3	3.03	21PS8085	0	100	6	6.00
21PS8106	2	98	3	3.06	22PS8004	0	100	6	6.00
20PS8077	2	98	3	3.06	20PS8009	0	100	6	6.00
21PS8090	3	97	3	3.09	21PS8024	1	99	6	6.06
21PS8087	4	96	3	3.12	22PS8001	1	99	6	6.06
23PS8033	0	100	4	4.00	GP6	1	99	6	6.06
GP3	0	100	4	4.00	20PS8004	1	99	6	6.06
20PS8048	0	100	4	4.00	21PS8012	2	98	6	6.12
22PS8022	1	99	4	4.04	23PS8011	2	98	6	6.12
GP1	1	99	4	4.04	20PS8097	2	98	6	6.12
20PS8041	1	99	4	4.04	22PS8020	3	97	6	6.19
23PS8026	2	98	4	4.08	23PS8006	3	97	6	6.19
17PS8019	2	98	4	4.08	20PS8059	3	97	6	6.19
GP5	2	98	4	4.08	21PS8081	4	96	6	6.25
20PS8020	2	98	4	4.08	20PS8022	4	96	6	6.25
23PS8020	3	97	4	4.12	21PS8094	5	95	6	6.32
20PS8015	3	97	4	4.12	21PS8101	10	90	6	6.67
23PS8019	4	96	4	4.17	21PS8017	1	99	7	7.07
20PS8084	4	96	4	4.17	21PS8020	1	99	7	7.07
22PS8008	6	94	4	4.26	23PS8013	1	99	7	7.07
21PS8058	7	93	4	4.30	22PS8012	2	98	7	7.14
21PS8138	7	93	4	4.30	23PS8009	2	98	7	7.14
22PS8010	7	93	4	4.30	GP7	2	98	7	7.14
20PS8116	0	100	5	5.00	22PS8006	4	96	7	7.29
21PS8010	1	99	5	5.05	GP2	6	94	7	7.45
23PS8003	1	99	5	5.05	20PS8018	6	94	7	7.45
20PS8001	1	99	5	5.05	20PS8067	2	98	8	8.16
20PS8114	1	99	5	5.05	21PS8086	9	91	8	8.79
20PS8026	2	98	5	5.10	21PS8041	0	100	9	9.00
20PS8053	2	98	5	5.10	20PS8118	4	96	11	11.46
21PS8111	3	97	5	5.15	21PS8043	1	99	12	12.12
20PS8012	3	97	5	5.15					

* Residual heterozygosity (%) was calculated as the proportion of heterozygous loci among valid SSR markers for each inbred line.
